# Risk of bleeding after abdominal paracentesis in patients with chronic liver disease and coagulopathy: A systematic review and meta‐analysis

**DOI:** 10.1002/jgh3.70013

**Published:** 2024-08-19

**Authors:** Jin Lin Tan, Thomas Lokan, Mohamed Asif Chinnaratha, Martin Veysey

**Affiliations:** ^1^ Faculty of Health and Medical Sciences The University of Adelaide Adelaide South Australia Australia; ^2^ Department of Gastroenterology and Hepatology Lyell McEwin Hospital Elizabeth Vale South Australia Australia; ^3^ Department of Gastroenterology Top End Health Service Darwin Northern Territory Australia; ^4^ School of Medicine Flinders University Bedford Park South Australia Australia

**Keywords:** abdominal paracentesis, ascitic drain, bleeding, chronic liver disease, coagulopathy

## Abstract

Abdominal paracentesis is a common procedure performed for both diagnostic and therapeutic purposes in patients with chronic liver disease and ascites. This review aims to provide an overview of the current evidence on the risk of bleeding associated with abdominal paracentesis. Electronic search was performed using PubMed, MEDLINE, and Ovid EMBASE from inception to 29 October 2023. Studies were included if they examined the risk of bleeding post‐abdominal paracentesis or the efficacy of interventions to reduce bleeding in patients with chronic liver disease. Random‐effects model was used to calculate the pooled proportions of bleeding events following abdominal paracentesis. Heterogeneity was determined by *I* ^2^, τ^2^ statistics, and *P*‐value. Eight studies were included for review. Six studies reported incident events of post‐abdominal paracentesis bleeding. Pooled proportion of bleeding events following abdominal paracentesis was 0.32% (95% CI: 0.15–0.69%). The mean values for pre‐procedural INR and platelet count of patients in these studies ranged between 1.4 and 2.0, and 50 and 153 × 10^9^/L, respectively. The highest recorded INR was 8.7, and the lowest platelet count was 19 × 10^9^/L. Major bleeding after abdominal paracentesis occurred in 0–0.97% of the study cohorts. Two studies demonstrated that the use of thromboelastography (TEG) before paracentesis in patients with chronic liver disease identified those at risk of procedure‐related bleeding and reduced transfusion requirements. The overall risk of major bleeding after abdominal paracentesis is low in patients with chronic liver disease and coagulopathy. TEG may be used to predict bleeding risk and guide transfusion requirements.

## Graphical abstract

The risk of major bleeding after abdominal paracentesis is low 0.32% (95% CI: 0.15‐0.69%) in patients with chronic liver disease or coagulopathy.

Viscoelastic tests may be useful as a predictor of bleeding or guide transfusion requirements prior to abdominal paracentesis.

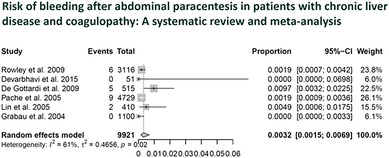

## Introduction

Abdominal paracentesis is a procedure commonly performed to remove ascitic fluid from the peritoneal cavity for diagnostic or therapeutic purposes. Most commonly, excess ascitic fluid accumulates in patients with chronic liver disease, malignancy, or heart failure.^1^, ^2^ However, the potential risk of bleeding associated with the procedure is a concern. Patients with chronic liver disease are at increased risk of developing both ascites and coagulopathy, further increasing the risk of major bleeding.^3^

Although conventional blood tests such as the International Normalized Ratio (INR) and platelet count are commonly performed before abdominal paracentesis, these investigations do not accurately reflect the actual state of coagulopathy in patients with chronic liver disease.^4^, ^5^, ^6^ The optimal strategy to risk‐stratify and prevent bleeding associated with abdominal paracentesis remains unclear.

This review paper aims to clarify the current evidence regarding the risk of bleeding after abdominal paracentesis in patients with coagulopathy, especially in those with chronic liver disease. Additionally, it seeks to investigate strategies for mitigating this risk.

## Methods

This study followed the preferred reporting items for Systematic reviews and Meta‐analyses (PRISMA).^7^ The study protocol was registered with the International Platform of Registered Systematic Review and Meta‐analysis Protocols (Registration ID: INPLASY202390062).^8^

### Eligibility criteria

Studies were selected if they reported any bleeding complications of abdominal paracentesis, including any investigations or management such as the measurement of platelet count, INR or thromboelastography, use of blood products, or ultrasound‐guided abdominal paracentesis. To be included in the systematic review, studies had to provide sufficient details for descriptive statistics (incident events of bleeding complications, the total number of abdominal paracenteses performed, mean INR, mean platelet count, or thromboelastography parameters). We excluded studies of children (<18 years), animals, non‐English publications, case reports, or abstracts.

### Search strategy

An electronic search was performed on clinical databases Pubmed Medline and Ovid EMBASE from database inception to 29 October 2023 using the following MeSH terms or free text: “ascites,” “paracentesis,” “ascitic drain,” “INR,” “international normalised ratio,” “coagulopathy,” “platelet,” and “thrombocytopaenia” (complete search strategies in Tables S1 and S2, Supporting information). Two independent reviewers (J.T. and T.L.) selected relevant studies based on the eligibility criteria. Titles and abstracts were screened to exclude studies that did not address the research questions. Subsequently, full texts were obtained for the remaining studies and assessed for eligibility. Any discrepancies were resolved by consensus between the two reviewers or discussion with a third senior author (M.V).

### Data collection and study quality assessment

The following data were extracted from each study: author, year, country, study type, participants, use of ultrasound, incident cases of bleeding, number of paracentesis performed, mean INR, and mean platelet count. Two investigators (J.T. and T.L.) extracted the data independently. Any discrepancies were resolved by consensus between the two reviewers or discussion with a third senior author (M.V.).

Study quality was assessed using a modified Newcastle–Ottawa Quality Assessment Scale (NOS) of nonrandomized studies.^9^ This assessment scale scores each study based on participant selection, comparability of groups, and measurements of outcome. A study can be awarded a maximum of four points under the “Selection” category. A maximum of one point can be given for “Comparability.” A maximum of three points can be awarded for “Outcomes.” Studies with modified NOS were rated high quality or moderate quality if the scores were greater than or equal to six and four, respectively, with the remaining rated as low quality.

### Statistical analysis

A random‐effects model, as described by DerSimonian and Laird, was used to calculate the pooled proportion of major bleeding events and 95% confidence intervals (CIs) following abdominal paracentesis among the included studies.^10^ The heterogeneity between studies was assessed using *I* ^2^ statistics and *P*‐value for the χ^2^ test of heterogeneity. An *I* ^2^ statistic of >50% or *P* < 0.05 implies significant heterogeneity. Publication bias was assessed using a funnel plot. All statistical analyses were performed using the “meta‐for” package on the R Project for Statistical Computing, developed by The R Foundation.^11^

## Results

### Search results

**Figure 1:**
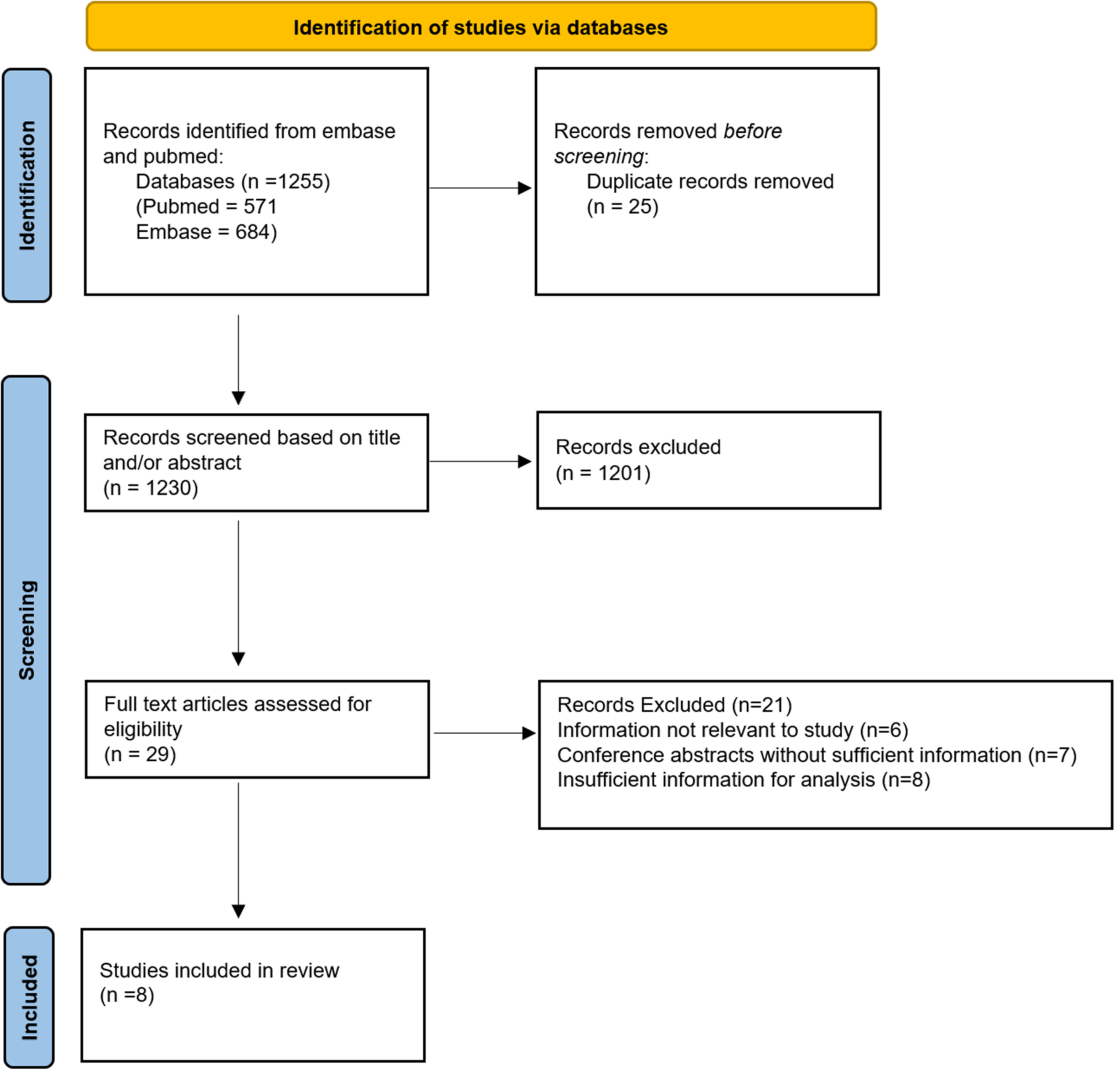
Modified PRISMA flow diagram of the search strategy and study selection.

There were 1255 studies identified by the initial search strategy, along with 25 duplicates, which were removed. From the initial screening of articles, 1230 were excluded based on the title and abstract not being relevant to this study. The remaining 28 articles were carefully assessed (by two assessors J.T. and T.L.) to ensure they met the criteria for the study. Of these 29 articles, 21 were eliminated for several reasons (not being fully relevant to the study, conference abstracts only, or containing insufficient data). The remaining eight articles met the inclusion criteria for this study, as shown in Figure 1.

### Study characteristics

Six studies reported events of post‐abdominal paracentesis bleeding.^12^, ^13^, ^14^, ^15^, ^16^, ^17^ Two studies explored the use of thromboelastography (TEG) before abdominal paracentesis in patients with chronic liver disease.^18^, ^19^ Across the included eight studies, a total of 9921 paracentesis procedures were performed in 1715 patients in the non‐TEG studies, and a further 213 procedures were performed in 140 patients in the TEG studies.^12^, ^13^, ^14^, ^15^, ^16^, ^17^, ^18^, ^19^ Overall, there were 45 individual episodes of clinically significant bleeding, with 22 in the non‐TEG studies and 23 in the TEG studies.^12^, ^13^, ^14^, ^15^, ^16^, ^17^, ^18^, ^19^ Study characteristics are summarized in Table 1. Based on the modified NOS, six of the included studies were of high quality,^12^, ^13^, ^14^, ^15^, ^18^, ^19^ and the remaining two studies were of moderate quality, as shown in Table 2.^18^, ^19^

**Table 1:** Cumulative incidence of post‐abdominal paracentesis bleed and associated mean INR and platelet count

Author and Year	Country/Regions	Study type	Clinical Cohort	Ultrasound	Incident cases/Total no. of abdominal paracentesis performed	%	Mean INR (SD)	Mean platelet × 10^9^/L (SD)
Rowley et al. 2009	United States	Retrospective	Chronic liver disease	Y	6/3116	0.19%	1.6 ± 0.6	121 ± 82
Devarbhavi et al. 2015	India	Retrospective	Budd–Chiari syndrome on anticoagulation INR 2–3	N	0/51	0%	3.02 ± 0.29	220 ± 190
De Gottardi et al. 2009	Europe	Prospective	Chronic liver disease	Y (11.7%)	5/515	0.97%	NM	NM
Pache et al. 2005	Canada	Retrospective	Chronic liver disease	Y (NM%)	9/4729	0.19%	2.0 ± 0.9	102 ± 37
Lin et al. 2005	Taiwan	Prospective	Ascites (26% non‐liver related diseases)	Y	2/410	0.48%	1.4 (SD not provided)	153 (SD not provided)
Grabau et al. 2004	United States	Prospective	Chronic liver disease	N	0/1100	0%	1.7 ± 0.46	50.4 (SD not provided)

CI, cumulative incidence, NM, not mentioned; SD, standard deviation.

**Table 2:** Modified Newcastle–Ottawa score of included studies

	Selection (maximum 4 points)			Comparability (maximum 1 point)	Outcomes (maximum 3 points)		Total (maximum 8 points)
	Representation (a) All patients diagnosed with chronic liver disease (1 point) (b) >90% of patients with chronic liver disease (1 point) (c) <90% of included patients has chronic liver disease (0 points) (d) No description (0 points)	Sample size (a) > =400 (1 point) (b) <400 (0 points)	Ascertainment of bleeding risk prior (a) INR and platelet +/− ROTEM (2 points) (b) INR or platelet or ROTEM (1 point) (c) No description (0 points)	Comparability investigates for potential confounders (1 point) Does not include confounders (0 points)	Assessment of outcome (a) Clinical and radiological evidence of bleeding (2 points) (b) clinical or radiological evidence of bleeding or clinical record (1 point) (c) No description (0 points)	Statistical test (a) Appropriate statistical methods (1 point) (b) Statistical test not appropriate, not described or incomplete (0 points)	
Rowley et al. 2019	1	1	2	1	2	1	8
Devarbhavi et al. 2015	1	0	2	1	1	0	5
De Gottardi et al. 2009	1	0	2	1	1	1	6
Pache et al. 2005	0	1	2	1	2	0	6
Lin et al. 2005	0	1	2	1	1	1	6
Grabau et al. 2004	1	1	2	0	1	0	5
Zanetto et al. 2021	1	0	2	1	2	1	7
De Pietri et al. 2016.	1	0	2	1	1	1	6

### Risk of bleeding after abdominal paracentesis

**Figure 2:**
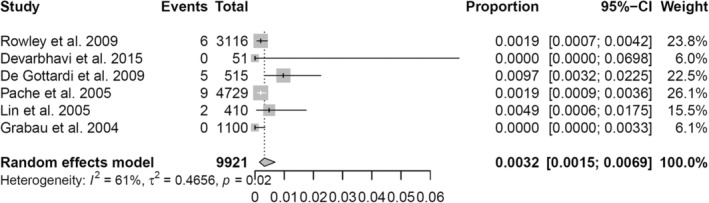
Forest plot—Pooled proportion of bleeding events following abdominal paracentesis.

**Figure 3:**
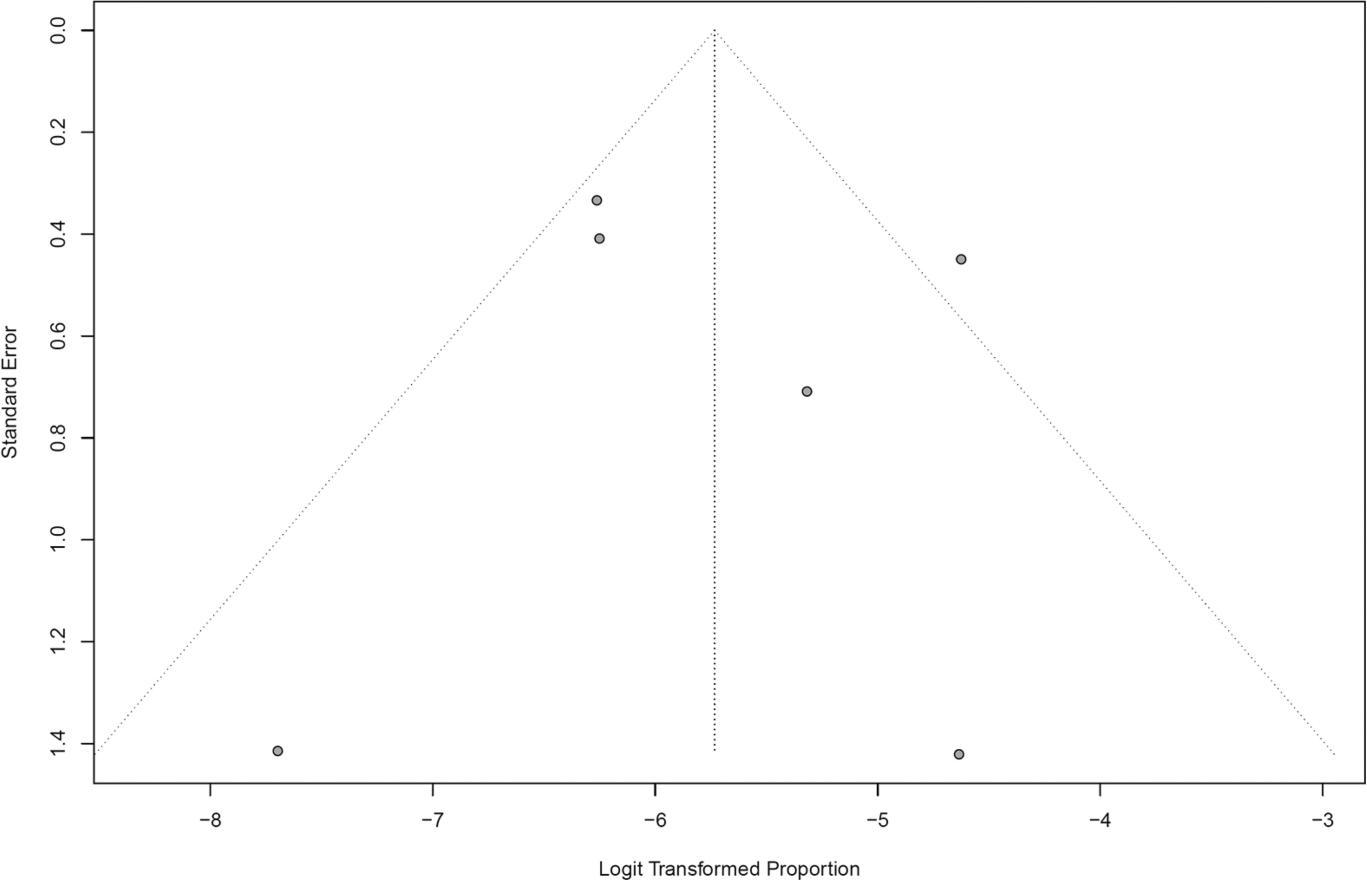
Funnel plot of included studies to assess publication bias.

Six studies reported the events of post‐abdominal paracentesis bleeding.^12^, ^13^, ^14^, ^15^, ^16^, ^17^ Clinically significant bleeding was generally defined as the presence of major abdominal wall hematoma or hemoperitoneum. The risk of major bleeding after abdominal paracentesis is low, with proportion ranging between 0 and 0.97% (Table 1). The pooled proportion of bleeding events following abdominal paracentesis was 0.32% (95% confidence interval: 0.15–0.69%), as demonstrated in Figure 2. There was significant heterogeneity among the included studies with a *I* ^2^ statistic of 61% and a *P*‐value of 0.02. The funnel plot of included studies appeared symmetrical, suggestive of a low risk of publication bias, as shown in Figure 3.

The highest recorded INR was 8.7, and the lowest platelet count was 19 × 10^9^/L. Excluding the study that included patients with Budd–Chiari syndrome on anticoagulation,^17^ the mean INR was between 1.4 and 2.0, and the mean platelet count was between 50 and 153 × 10^9^/L. Individual case analyses included in these papers showed that patients had bleeding complications despite normal INR of 1.0 or a platelet count greater than 150 × 10^9^/L.

### Thromboelastography

Two studies were explored using TEG before abdominal paracentesis in patients with chronic liver disease.^18^, ^19^

A pilot study by Zanetto and colleagues showed that TEG identified patients with decompensated cirrhosis who are at risk of procedure‐related bleeding. This prospective study included 72 patients with decompensated liver disease, seven of whom had procedure‐related bleeding. Conventional coagulation parameters such as INR and platelet count were comparable between bleeding and nonbleeding patients, whereas TEG parameters were all indicative of hypercoagulability: k‐time (4.5 min *vs* 2.2 min; *P* = 0.02), α‐angle (34° *vs* 59°; *P* = 0.003), and maximum amplitude (37 mm *vs* 50 mm; *P* = 0.004). However, a larger cohort of patients would be required to define a threshold to identify patients at risk of procedural‐related bleeding.^18^

De Pietri from Italy conducted a randomized controlled trial that demonstrated that TEG‐guided transfusion reduced blood product transfusion without increasing the risk of bleeding in patients with chronic liver disease. This study included 60 patients, with either INR >1.8 or platelet <50 × 10^9^/L, randomized to TEG‐guided transfusion or standard of care (SOC) strategy. The TEG group received fresh frozen plasma (FFP) if the “time to clot initiation” (i.e. reaction time) was more than 40 min or platelet transfusion if the “maximum amplitude” was more than 30 mm. The overall blood product requirement was significantly lower in the TEG‐guided transfusion *vs* SOC (16.7% *vs* 100%; *P* < 0.0001). One patient in the SOC group developed hemoperitoneum due to inadvertent injury to an abdominal vessel. This patient had received FFP prior, and bleeding was unlikely related to coagulopathy.^19^

## Discussion

The risk of bleeding from abdominal paracentesis in patients with chronic liver disease and coagulopathy has been a concern in clinical practice. Our review of the available literature indicates that the overall risk of major bleeding following abdominal paracentesis is low. The proportion of clinically significant bleeding, defined as the presence of major abdominal wall hematoma or hemoperitoneum, was 0.32% (95% confidence interval: 0.15–0.69%), or approximately 1 in 300, across the included studies. These findings suggest that the procedure is generally safe, even in patients with underlying coagulopathy.

Bleeding complications from abdominal paracentesis occurred in patients with normal INR of 1.0 and platelet counts greater than 150 × 10^9^/L. This observation is consistent with previous studies, which demonstrated that bleeding after invasive procedures is not accurately predicted by platelet counts or INR in cirrhotic patients.^20^, ^21^ However, it is worth noting that among the included studies, the mean INR was between 1.4 and 2.0, and the mean platelet count was between 50 and 153 × 10^9^/L. In addition, there were no reported cases of abdominal paracentesis performed on patients with platelet count of less than 19 × 10^9^/L or INR greater than 8.9. There are also other factors to be considered, such as renal function, MELD score, presence of sepsis, Child–Pugh score, operator experience, use of ultrasound guidance, and concurrent use of antiplatelet or anticoagulant medications, which could contribute to bleeding risk and should be considered in clinical decision‐making.^12^, ^13^, ^14^, ^15^, ^16^, ^17^, ^22^ Current guidelines from major societies recommend that clinicians should not routinely correct thrombocytopenia or coagulopathy before abdominal paracentesis.^23^, ^24^, ^25^

Thromboelastography (TEG) and rotational thromboelastography (ROTEM) have emerged as new tools to assess and manage coagulopathy more accurately than conventional blood tests such as platelet count and INR.^26^ In a systematic review by Wei et al., five randomized controlled trials demonstrated that TEG‐guided transfusion before an invasive procedure significantly reduced the overall blood product transfusion compared with the SOC in patients with cirrhosis and coagulopathy.^27^ However, there are limited data to show that TEG or ROTEM could predict risk of clinically significant bleeding post invasive procedures.^27^ While viscoelastic tests could not be generally indicated to predict bleeding risk, they can be used to assess the severity of disease or hemostatic status and to provide an initial benchmark to guide management in the case of post‐procedural bleeding.^28^ Given that abdominal paracentesis has a low risk of bleeding overall, TEG and ROTEM may only be helpful or cost‐effective in patients with a higher risk of bleeding. More research is required to identify and validate those factors and their target levels, which may have a role in the evaluation of clotting in patients with decompensated cirrhosis.^23^

To mitigate bleeding risks in abdominal paracentesis, current guidelines and position statements recommend using ultrasound guidance where available.^25^, ^29^ Individual case analyses of major bleeding in our included studies revealed that traumatic injury to abdominal vessels is a common reason for bleeding following abdominal paracentesis.^14^, ^15^, ^17^ Previous works have demonstrated in a retrospective study that ultrasound‐guided paracentesis results in near‐zero hemorrhage risk even without correction of coagulation abnormalities.^12^ A prospective, randomized, controlled trial comparing ultrasound for abdominal paracentesis showed a 95% success rate compared with control at only 61%.^30^ Ultrasound usage reduces the bleeding risk by providing operators with information such as the volume and location of intraperitoneal free fluid to guide clinical decision‐making on whether paracentesis can be safely performed. Color Doppler ultrasound has the additional advantage of identifying and avoiding abdominal wall blood vessels.^29^ Therefore, clinicians should consider using ultrasound guidance, if available, when performing abdominal paracentesis.

In the absence of ultrasound, operator skill is vital in minimizing bleeding risk. Among the studies we reviewed, one study proposed a graduated system of supervision leading from direct supervision of operators without physician intervention to demonstrate competence (at least three paracenteses), and finally independent performance without supervision. Of the 1100 paracenteses performed by these operators, no significant bleeding events (0%) occurred in this study despite a wide range of platelet counts (19 × 10^9^/L to 341 × 10^9^/L) and INR values (0.9–8.7). This highlights the importance of good procedural techniques to avert major bleeding complications when ultrasound is unavailable.

This systematic review has several key strengths. First, this study included over 10 000 abdominal paracenteses to investigate the risk of bleeding associated with this procedure in patients with coagulopathy. The included studies encompass a diverse cohort of patients from various geographical locations. Many of the studies accounted for confounding factors such as chronic disease, Model for End‐stage Liver Disease (MELD) scores, and Child–Pugh scores. This comprehensive analysis provides valuable insights into the relationship between coagulopathy and the risk of bleeding during abdominal paracentesis, particularly in patients with chronic liver disease and ascites.

There are several limitations to this review. First, there were significant heterogeneity among the included studies due to differences in the included cohort, use of ultrasound, and variations in the definition of clinically significant bleeding. Second, the bleeding risks that were reported combined both diagnostic and therapeutic abdominal paracentesis, for which the latter may have a higher risk of bleeding. Third, the limited number of studies that utilized TEG restricts the strength of the results regarding its use in predicting bleeding risk during abdominal paracentesis. Further research incorporating TEG, including prospective studies and cost‐effectiveness analysis, will provide more robust evidence. Fourth, although ultrasound guidance is widely used in clinical practice to improve the safety and accuracy of abdominal paracentesis, none of the studies had adjusted for the effects of ultrasound as a possible confounder of bleeding related to abdominal paracentesis. Lastly, other potential risk factors, such as non‐vitamin K antagonist oral anticoagulants (NOACs) or direct‐acting oral anticoagulants (DOACs), were not consistently addressed in the included studies.

## Conclusion

In conclusion, our review suggests that abdominal paracentesis is a safe procedure, even in patients with coagulopathy. However, bleeding risk is multifactorial, and traditional coagulation tests may not always predict complications. TEG shows promise as a potential tool for risk assessment and individualized patient management. Further research is required to refine our understanding of bleeding risk in this patient population.

## Supporting information


**Table S1.** Embase search strategies and results.
**Table S2.** Pubmed search strategies and results.
