# Supplementary material for: Risk of bleeding after abdominal paracentesis in patients with chronic liver disease and coagulopathy: A systematic review and meta‐analysis
Source: JGH Open. 2024 Aug 19;8(8):e70013. doi: 10.1002/jgh3.70013 (PMC11331248; doi:10.1002/jgh3.70013)
Supplement: Supplementary file 1 — Table S1. Embase search strategies and results. Table S2. Pubmed search strategies and results. [file JGH3-8-e70013-s001.docx]

## Supplementary Data

### Search Strategy

**Table 1: Embase search strategies and results**

| **#** | **Query** | **Results up to 29th Sep 2022** |
| --- | --- | --- |
| 1 | ascites fluid/ or ascites/ | 61,322 |
| 2 | drain/ or peritoneal drain/ or paracentesis/ | 18,049 |
| 3 | 1 and 2 | 4,950 |
| 4 | [inr.mp](http://inr.mp/). or international normalised ratio/ | 49,906 |
| 5 | [coagulopathy.mp](http://coagulopathy.mp/). or blood clotting disorder/ | 50,089 |
| 6 | 4 or 5 | 95,309 |
| 7 | 3 and 6 | 407 |

##

**Table 2: Pubmed search strategies and results**

| **No.** | **Query** | **Search Details** | **Results up to 29 Sep 2022** |
| --- | --- | --- | --- |
| 1 | ascites[MeSH Terms] | "ascites"[MeSH Terms] | 17,487 |
| 2 | paracentesis[MeSH Terms] | "paracentesis"[MeSH Terms] | 12,846 |
| 4 | (paracentesis[MeSH Terms]) OR (ascitic drain[MeSH Terms]) | "paracentesis"[MeSH Terms] OR (("ascite"[All Fields] OR "ascites"[MeSH Terms] OR "ascites"[All Fields] OR "ascitic"[All Fields]) AND "drainage"[MeSH Terms]) | 13,889 |
| 5 | (INR[MeSH Terms]) OR (international normalised ratio[MeSH Terms]) | "international normalized ratio"[MeSH Terms]" | 6,025 |
| 6 | coagulopathy[MeSH Terms] | "blood coagulation disorders"[MeSH Terms] | 103,572 |
| 7 | #1 OR #2 OR #4 | "ascites"[MeSH Terms] OR "paracentesis"[MeSH Terms] OR ("paracentesis"[MeSH Terms] OR (("ascite"[All Fields] OR "ascites"[MeSH Terms] OR "ascites"[All Fields] OR "ascitic"[All Fields]) AND "drainage"[MeSH Terms])) | 30,070 |
| 8 | #5 OR #6 | "international normalized ratio"[MeSH Terms] OR "international normalized ratio"[MeSH Terms] OR "blood coagulation disorders"[MeSH Terms] | 109,160 |
| 9 | #7 AND #8 | ("ascites"[MeSH Terms] OR "paracentesis"[MeSH Terms] OR ("paracentesis"[MeSH Terms] OR (("ascite"[All Fields] OR "ascites"[MeSH Terms] OR "ascites"[All Fields] OR "ascitic"[All Fields]) AND "drainage"[MeSH Terms]))) AND ("international normalized ratio"[MeSH Terms] OR "international normalized ratio"[MeSH Terms] OR "blood coagulation disorders"[MeSH Terms]) | 269 |
